# Women Veterans’ Healthcare Needs, Utilization, and Preferences in Veterans Affairs Primary Care Settings

**DOI:** 10.1007/s11606-022-07585-3

**Published:** 2022-08-30

**Authors:** Kate L. Sheahan, Karen M. Goldstein, Claire T. Than, Bevanne Bean-Mayberry, Catherine C. Chanfreau, Megan R. Gerber, Danielle E. Rose, Julian Brunner, Ismelda A. Canelo, Jill E. Darling MSHS, Sally Haskell, Alison B. Hamilton, Elizabeth M. Yano

**Affiliations:** 1 JSI, Inc., 2733 Crystal Dr 4th floor, Arlington, VA 22202 USA; 2 Division of General Internal Medicine, Duke University School of Medicine, Durham, NC USA; 3 VA HSR&D Center for the Study of Healthcare Innovation, Implementation and Policy, VA Greater Los Angeles Healthcare System, Los Angeles, CA USA; 4 Department of Medicine, University of California, Los Angeles (UCLA) Geffen School of Medicine, Los Angeles, CA USA; 5 Veterans Affairs Informatics and Computing Infrastructure (VINCI), Salt Lake City, UT USA; 6 Albany Stratton VA Medical Center, Albany, NY USA; 7 Division of General Internal Medicine, Albany Medical College, Albany, NY USA; 8 Center for Economic and Social Research (CESR), University of Southern California, Los Angeles, CA USA; 9 Pain Research, Informatics, Multi-morbidities, and Education (PRIME) Center, VA Connecticut Healthcare System, West Haven, CT USA; 10 Division of General Internal Medicine, Department of Medicine, Yale University School of Medicine, West Haven, CT USA; 11 Office of Women’s Health, Veterans Health Administration, Washington, DC USA; 12 Department of Psychiatry and Biobehavioral Sciences, UCLA Geffen School of Medicine, Los Angeles, CA USA; 13 Department of Health Policy & Management, UCLA Fielding School of Public Health, Los Angeles, CA USA

**Keywords:** primary care, mental health, women’s health, Veterans, trauma

## Abstract

**Background:**

The Veterans Health Administration (VA) is the largest integrated health system in the US and provides access to comprehensive primary care. Women Veterans are the fastest growing segment of new VA users, yet little is known about the characteristics of those who routinely access VA primary care in general or by age group.

**Objective:**

Describe healthcare needs, utilization, and preferences of women Veterans who routinely use VA primary care.

**Participants:**

1,391 women Veterans with 3+ primary care visits within the previous year in 12 VA medical centers (including General Primary Care Clinics, General Primary Care Clinics with designated space for women, and Comprehensive Women’s Health Centers) in nine states.

**Methods:**

Cross-sectional survey (45% response rate) of sociodemographic characteristics, health status (including chronic disease, mental health, pain, and trauma exposure), utilization, care preferences, and satisfaction. Select utilization data were extracted from administrative data. Analyses were weighted to the population of routine users and adjusted for non-response in total and by age group.

**Key Results:**

While 43% had health coverage only through VA, 62% received all primary care in VA. In the prior year, 56% used VA mental healthcare and 78% used VA specialty care. Common physical health issues included hypertension (42%), elevated cholesterol (39%), pain (35%), and diabetes (16%). Many screened positive for PTSD (41%), anxiety (32%), and depression (27%). Chronic physical and mental health burdens varied by age. Two-thirds (62%) had experienced military sexual trauma. Respondents reported satisfaction with VA women’s healthcare and preference for female providers.

**Conclusions:**

Women Veterans who routinely utilize VA primary care have significant multimorbid physical and mental health conditions and trauma histories. Meeting women Veterans’ needs across the lifespan will require continued investment in woman-centered primary care, including integrated mental healthcare and emphasis on trauma-informed, age-specific care, guided by women’s provider preferences.

## INTRODUCTION

The population of women Veterans within the Veterans Health Administration (VA) is growing rapidly, with important implications for primary care delivery. While a numerical minority of all users, the absolute number of women Veterans utilizing VA services is increasing more swiftly than that of men Veterans.^1^ Between 2000 and 2015, the number of women Veterans accessing VA primary care almost tripled to nearly 450,000. Women Veterans thus constitute an increasing share of all VA users, growing from 5% of total users in 2000 to 8% in 2015 with a projected increase to 11% by 2024.^2,3^ The women Veteran population using VA is also aging. Their age distribution is trimodal, with three peaks at ages 32, 53, and 91.^1^ These peaks represent different life stages, each with specific healthcare needs.

Given the historically lower enrollment of women in the military, the VA healthcare delivery system has not needed the staffing and resources to provide comprehensive care for a large population of women Veterans. However, as their numbers have risen, VA has invested heavily in program development and evaluation, redesigned service delivery, provider training, and infrastructure to improve women Veterans’ access to high-quality care.^4^ In 2010, VA established minimum women’s health service delivery requirements and laid the groundwork for provision of primary care at one location from a single provider with the clinical proficiencies to meet women’s needs.^5^ VA facilities now offer primary care services to women through one or more of the following models: mixed gender General Primary Care Clinics, Comprehensive Primary Care Clinics with designated space for women, and Comprehensive Women’s Clinics. Many facilities offer Comprehensive Primary Care Clinics with designated space for women, and Comprehensive Women’s Clinics in addition to General Primary Care Clinics. In 2020, Congress passed the *Deborah Sampson Act* to further close women’s healthcare gaps and advance research.^6^

Despite significant advances and investment in VA women’s care, women may still face access barriers, lack of continuity, and care coordination challenges when using VA services.^7–10^ A recent study highlighted limited availability of women’s health providers and services, inefficient referral processes for community-based care, harassment at VA facilities, and difficulty scheduling appointments.^11^ Although optimizing women-centered primary care has been a key part of VA’s efforts to address these issues, relatively little is known about the health status, utilization, preferences, or satisfaction of women Veterans who routinely use VA primary care. We used a multi-state practice-based survey of women Veterans who routinely use VA primary care to address this knowledge gap and inform evidence-based improvements in care for women generally and by age group.

## METHODS

We used baseline data collected for a cluster randomized controlled trial that assessed an evidence-based quality improvement approach to tailor VA’s patient-centered medical home model (Patient Aligned Care Teams) to better meet women’s needs. Detailed study methods are reported elsewhere.^12^ The study recruited from 12 VA medical centers in nine states spanning four VA regional networks. The sites offered services through a mix of care models (two facilities with mixed gender General Primary Care Clinics, two facilities with General Primary Care Clinics and Comprehensive Primary Care Clinics with designated space for women, 1 facility with a Comprehensive Women’s Clinic, and 7 facilities with General Primary Care Clinics and Comprehensive Women’s Clinics). We defined women with 3+ visits in the previous year as “routine users” because three is the average annual number of primary care encounters among women VA users nationally.^1^ Using VA administrative data, we identified 4,307 women Veterans at participating facilities who had 3+ primary or women’s healthcare visits in the previous year. Participants may have also accessed non-VA care. At four rural sites with fewer eligible patients, all were selected for participation, while at the other, larger VA facilities participants were randomly selected. Among these women, 3,102 were eligible for interview and 1,395 completed a telephone survey using computer-assisted telephone interviewing (CATI) between January and March 2015 (response rate 45%). The CATI survey captured data for all measures except for utilization of VA mental health, primary care-mental health integrated care (PC-MHI), emergency room, and hospitalization. This information was extracted from the VA Corporate Data Warehouse (CDW), where all VA patient encounter data are stored. Women’s primary care model availability at each facility was assessed using Women’s Assessment Tool for Comprehensive Health (WATCH) survey data. WATCH assesses availability and delivery of comprehensive care for women Veterans at each VA primary care site annually.^13^ The study was approved by the VA Institutional Review Board.

### Measures

#### Sociodemographic Characteristics

We collected age, race, ethnicity, sexual orientation, gender identity, marital status, number of children in household, education, employment, and health insurance status.

#### Physical Health

We collected information on physical and mental conditions, experiences, and behaviors shown to have a substantial influence on Veteran health and primary care services delivery.^14–19^ For each woman, we calculated the Seattle Index of Comorbidity (SIC), which captures patient-reported age, smoking status, and seven chronic medical conditions (pneumonia, diabetes, cancer, lung disease, heart attack, stroke, congestive heart failure).^20^ Scores range from 0 to 20–25 (age dependent); higher scores indicate more severe comorbidity.

#### Mental Health and Trauma

We used validated screeners for depression (Patient Health Questionnaire 2 or PHQ-2), anxiety (Generalized Anxiety Disorder 2 scale or GAD2), and the Posttraumatic Stress Disorder Checklist (PTSD Checklist or PCL).^21–23^ Self-rated overall health and pain were measured with the Veterans RAND 12-item Health Survey. ^24^ Alcohol use was measured with the Alcohol Use Disorders Identification Test-Concise (AUDIT-C). ^25^ Scores range from 0 to 12; 3+ indicates disorder. Military sexual trauma (MST) was measured using the VA’s two-item screener, which assesses sexual harassment, unwanted sexual contact, and sexual coercion while in the military.^26^ Combat exposure was defined as having ever served in a combat or war zone, received hostile fire, or seen someone severely wounded or killed during military service.

#### Healthcare Utilization, Preferences, and Satisfaction

Utilization of VA and non-VA healthcare was assessed by asking sources of overall healthcare, women’s health, mental health, and specialty care in the last 12 months. We extracted prior year VA utilization information from VA CDW for mental health, primary care-mental health integrated care (PC-MHI), emergency room, and hospitalization.

We asked women’s preference for a male or female provider when accessing basic primary care (e.g., routine care for illnesses) and women-specific care (e.g., pelvic exams). Women rated the importance of having women-only health clinics at VA on a 4-point scale (1 = not at all important, 4 = extremely important).

Seven items from the Primary Care Satisfaction Survey for Women were used to measure satisfaction with VA provider knowledge and skill related to women-specific health issues.^27^ Responses ranged from strongly disagree to strongly agree and were averaged and then transformed onto a 0–100 scale, with higher scores indicating greater satisfaction. The survey queried whether women would recommend VA care to others and whether they perceived VA women’s healthcare as better, the same, or worse than care provided outside VA.

### Analysis

We conducted descriptive analysis for the overall sample and by age group. From the 1,395 patients who completed the survey, we excluded 4 with missing age information, for an analytical sample of 1,391. All analyses are weighted to the population of women routine users of VA primary care, and for sample design and to address potential non-response bias. Analyses were conducted using Stata Version 15 (College Station, TX: StataCorp LLC).

## RESULTS

We report overall findings and findings by age. Mean age was 49.8 (± 14.3) years (Table 1). Most, 59%, were non-Hispanic White; 28% were non-Hispanic Black. The majority, 62%, were not married. Ten percent reported their sexual orientation as gay or bisexual and 2% reported transgender identity. Overall, 40% had a college or graduate degree and 44% were employed. More than half, 57%, had health insurance from a non-VA source.

**Table 1:** Demographic Characteristics of Women Veterans Who Routinely Use VA Primary Care*

Characteristic	Weighted % (*N*= 1,391)
Age	
18–44	35.0
45–64	50.5
65+	14.5
Race/ethnicity	
Hispanic	6.8
Non-Hispanic White	59.0
Non-Hispanic Black	28.2
Non-Hispanic Asian	0.9
Non-Hispanic other	1.6
Multiple	6.4
Marital status	
Married/partner	37.6
Divorced/widowed	39.3
Never married	23.1
Children in household	
None	74.8
Children <18	25.2
Sexual orientation/identity	
Non-LGBT	85.2
Lesbian/gay	7.3
Bisexual	3.0
Transgender	2.1
Highest level of education completed	
High school	16.8
Some college	43.6
College or graduate degree	39.6
Health insurance coverage^†^	
VA only	42.7
Medicare/Medicaid	23.8
Private/employer	20.2
CHAMP/Tri-Care/other military	7.0
Other	6.4
Employment	
Employed	43.9
Unemployed	5.7
Not in labor force	50.4

Notes: *Routine VA primary care users were defined as someone who accessed primary care or women-specific services at VA at least 3 times in the year prior to the survey. ^†^Categories other than “VA only” are not exclusive

### Physical Health

On average, 37% considered themselves in poor or fair health (Table 2). Overall, 72% reported at least one chronic condition, with the lowest prevalence among women aged 18–44 (45%) and the highest among women aged 65+ (96%). Hypertension and elevated cholesterol were the most common conditions. Median SIC score was two among women aged 18–44 and seven among women 65+. Pain interfered with normal work “a lot” or “all the time” for 35% of women, with interference highest (43%) among women aged 45–64. More than 20% of women Veterans scored 3+ on the AUDIT-C; the highest percentage (26%) was among women aged 18–44. Similarly, almost a quarter of all women identified as current smokers, with the highest percentage (26%) among women aged 45–64.

**Table 2:** Health Status of Women Veterans Who Routinely Use VA Primary Care*

Health status	Weighted %			
	All	Age 18–44	Age 45–64	Age 65+
	*N*=1,391	*N*=370	*N*=756	*N*=265
Overall health (self-reported)				
Poor	8.0	6.7	9.7	5.3
Fair	29.2	29.8	26.4	26.4
Good/very good	58.3	58.5	57.7	59.2
Excellent	4.6	5.3	2.8	9.1
Chronic conditions (self-reported)				
Hypertension	42.3	17.6	49.9	75.7
Elevated cholesterol/hyperlipidemia	39.2	15.3	49.4	61.7
Osteoporosis	18.8	3.4	22.1	45.6
Pneumonia	18.8	15.2	19.8	23.7
Diabetes	16.1	5.3	20.9	25.1
Cancer	12.3	6.4	12.7	25.7
Lung disease	9.1	2.4	11.8	16.0
Heart attack	4.5	1.7	4.3	11.9
Stroke	3.9	0.6	4.6	9.4
Congestive heart failure	2.9	0.2	2.9	9.7
Total chronic conditions				
0	28.4	55.5	16.9	3.5
1	25.5	28.4	27.1	12.4
2–4	40.4	15.8	49.9	66.2
5+	5.8	0.4	6.1	17.9
Seattle Index of Comorbidity^†^				
Mean ± SD	3.5 ±2.9	1.7 ± 1.6	3.6 ± 2.6	7.4 ± 3.3
Median	4.0	2.0	4.0	7.0
Range	0–18	0–7	0–12	3–18
Extent to which pain interferes with normal work				
A lot/all the time	35.1	25.2	42.7	31.1
A little/somewhat	40.7	44.8	39.1	37.1
Not at all	24.2	30.1	18.2	31.8
Mental health screens (% positive)				
PTSD	41.2	43.2	44.5	24.1
Depression (PHQ2)	27.2	27.2	30.0	16.1
General anxiety (GAD2)	31.9	35.9	33.5	15.2
Had ≥ 1 positive mental health screen^‡^	48.9	53.9	50.3	31.2
Had ≥ 1 positive mental health screen and one chronic illness^§^	36.0	26.2	44.0	30.6
Military sexual trauma (MST)	61.5	60.2	68.0	40.7
Lifetime sexual trauma				
Before 18 years old	40.0	38.4	44.4	26.4
After 18 and before military	24.2	21.3	28.0	17.6
After military	25.8	23.5	28.7	20.5
Combat exposure	51.1	70.1	40.8	41.8
AUDIT-C score 3+^‖^	22.1	26.0	21.0	16.0
Smoking status				
Current smoker	23.2	21.6	26.4	14.6
Past smoker	31.1	20.6	35.2	42.8
Never smoked	45.7	57.8	38.4	42.6

Notes: *Routine VA primary care users were defined as someone who accessed primary care or women-specific services at VA at least 3 times in the year prior to the survey. ^†^ Includes self-reported diagnosis of one or more of the chronic conditions listed under “Chronic conditions (self-reported).” ^‡^ Includes PTSD, depression, and anxiety. ^§^ Includes PTSD, depression, or anxiety and one or more of the chronic conditions listed under “Chronic conditions (self-reported).” ^‖^*AUDIT-C*, Alcohol Use Disorders Identification Test-Concise

### Mental Health and Trauma

Mental health comorbidities and experiences that may engender them, including sexual trauma and combat exposure, were common (Table 2). Half had at least one positive mental health screen; the percentage of positive screens was lowest (31%) among women 65+. Overall, 40% screened positive for symptoms of PTSD, 27% for depression, and 32% for anxiety. More than one-third reported at least one positive mental health screen *and* one chronic illness; mental and physical health comorbidity was highest (45%) among women aged 45–64. Experience of sexual trauma was common; 40% reported childhood sexual trauma and 62% reported MST. Women aged 45–64 reported the highest prevalence of childhood sexual trauma (44%) and MST (68%). Half of the women reported combat exposure; women aged 18–44 had the highest rate (70%).

### Healthcare Utilization, Preferences, and Satisfaction

Overall, 62% of women routine users of VA primary care accessed all their healthcare in the VA (Table 3). Almost 80% had accessed specialty care exclusively from VA in the previous year. Across age groups, women Veterans had a median of two emergency room visits and one hospitalization at VA in the prior year. Just over half of women (57%) utilized mental or behavioral health services in the year prior to the survey. The percentage of women accessing mental or behavioral health services was approximately 60% among women aged 18–64 and lower (36%) among women 65 years and older. The median number of prior year mental health visits was six for women aged 65+ and eight for women in the younger age groups.

**Table 3:** Healthcare Preferences, Utilization, and Satisfaction of Women Veterans Who Routinely Use VA Primary Care*

	Weighted %			
	All	Age 18–44	Age 45–64	Age 65+
	*N*=1,391	*N*=370	*N*=756	*N*=265
Importance of women’s healthcare from a women-only provider or clinic				
Not at all important	12.0	12.1	10.8	16.2
Somewhat/moderately important	36.7	45.6	30.1	39.6
Extremely important	50.2	42.3	58.1	40.6
Preference for female providers				
For women-specific health services	70.0	66.8	73.2	65.8
For basic primary care services	47.6	40.8	53.6	42.3
Source of overall healthcare				
VA only	62.0	60.5	62.4	64.7
Dual use of VA and non-VA	38.2	40.8	37.5	34.2
Model of care offered at facility attended	6.6			
General primary care clinic	15.9	-	-	-
General Primary Care Clinic + separate designated space	72.5	-	-	-
General Primary Care Clinic + Comprehensive Women’s Health Center	15.9	-	-	-
Comprehensive Women’s Health Center	5.0	-	-	-
Use of VA or non-VA specialty care in past 12 months				
VA only	77.5	79.2	78.1	70.7
Non-VA only	6.7	5.7	6.6	9.9
Dual use VA and non-VA	0.1	0	0	0.4
No specialty care in past 12 months	15.8	15.2	15.3	19.0
Use of women’s health services in past 12 months				
VA only	90.7	90.6	92.2	85.3
Non-VA only	5.8	6.2	4.5	9.2
Dual use VA and non-VA	1.8	2.3	1.8	0.7
No women’s healthcare in past 12 months	1.8	0.88	1.6	4.8
Use of mental health or behavioral healthcare in past 12 months				
VA only	43.9	45.7	46.6	29.7
Non-VA only	2.2	3.8	1.4	0.7
Dual use VA and non-VA	10.5	11.9	11.2	4.2
Other	0.5	0.3	0.6	1.0
No mental healthcare in past 12 months	42.9	38.2	40.2	64.4
Emergency room visits				
Mean ± SD (weighted)	2.3 ± 2.5	2.2 ± 2.4	2.3 ± 2.3	2.5 ± 3.1
Median	2.0	1.0	2.0	2.0
Hospitalization				
Mean ± SD (weighted)	1.8 ± 1.3	1.7 ± 1.3	1.7 ± 1.1	2.3 ± 1.8
Median	1.0	1.0	1.0	1.0
Mental health visits^†^				
Mean ± SD (weighted)	17.4 ± 31.0	17.6 ± 26.6	18.3 ± 34.6	11.3 ± 16.5
Median	8.0	8.0	8.0	6.0
PC-MHI visits^†‡^				
Mean ± SD (weighted)	6.4 ± 7.7	5.6 ± 6.7	7.0 ± 8.2	6.1 ± 7.6
Median	4.0	4.0	4.0	4.0
Patient satisfaction with provider score				
Mean ± SD (weighted)	86.5 ± 19.8	85.0 ± 17.6	86.4 ± 21.2	90.8 ± 16.7
Median	96.4	92.9	96.4	96.4
Would patient recommend VA care to other women Veterans				
Strongly recommend	58.6	50.1	62.0	67.4
Recommend	32.2	36.4	30.5	28.3
Not recommend/strongly not recommend	6.0	9.3	5.0	1.9
Recommend some aspects of VA but not others/neutral	3.1	4.3	2.5	3.1
Patient assessment of women’s care provided in VA compared to women’s care provided outside VA				
VA care is better	26.6	25.5	26.0	31.8
Same	32.5	31.5	32.6	35.0
Outside care is better	20.0	30.0	17.4	6.3
Not sure	20.8	13.4	24.1	26.9

Notes: *Routine VA primary care users were defined as someone who accessed primary care or women-specific services at VA at least 3 times in the year prior to the survey. ^†^Some overlap may exist between mental health and PC-MHI visits. ^‡^*PC-MHI*, primary care/mental health integrated visit

Across age groups, approximately 80–90% rated having a women-only provider/clinic as moderately-to-extremely important. Most women (70%) prefer female providers for women-specific care, while approximately half prefer female providers for general primary care. The mean patient satisfaction with provider score was 86.5/100 (Table 3). The vast majority (91%) would recommend VA to other women Veterans. While 60% considered women’s care provided in VA the same or better than care provided outside VA, 20% perceived outside care as better; this perception was most prevalent (30%) among the youngest age group.

## DISCUSSION

We found that women Veterans who routinely use VA primary care and women’s health services have complex physical and mental health multi-morbidities, and that these vary by age in marked ways. Sexual trauma, during childhood and/or military service, was pronounced among nearly two-thirds of routine users, and combat exposure was also common. High healthcare utilization mapped to the level of self-reported healthcare need. Overall, women Veterans preferred women providers and women-only care environments, and they rated their VA care highly.

Our results, aligning with those of Lehavot et al. (2012), show higher prevalence of elevated cholesterol, diabetes, and cancer than among the general population of women. ^28–31^ Our results also show higher tobacco and alcohol use, both associated with trauma exposure and increased chronic illness.^32–34^ Consistent with Naylor (2019) and others, we found high prevalence of pain interference with daily living.^35,36^ Such chronic pain is correlated with functional impairment (e.g., involuntary unemployment, disability) and psychiatric pathologies.^16,37^ The prevalence of pain therefore requires continued efforts within primary care to routinely assess pain and link patients to multidisciplinary pain management interventions

Our findings reinforce prior studies showing extensive mental health needs and trauma exposure among women Veterans.^17^ Previous research shows that women Veterans have higher mental health comorbidity than non-Veteran women and that women generally have higher mental health comorbidity than men.^31,38–40^ Among women Veterans who routinely use VA primary care, childhood and military sexual trauma is a common experience that is associated with increased frequency and severity of psychological issues, alcohol use, impaired health status, chronic illness, and chronic pain.^41–47^ Additionally, our results show that a substantial percentage of women Veterans who routinely use VA primary care, particularly those aged 18–44, have experienced combat, which is linked to PTSD, depression, tobacco use, and alcohol misuse.^34,48–50^ While this study did not assess interpersonal violence, also related to adverse mental and physical health sequelae, other studies highlight its frequency among women Veterans, particularly those who utilize VA care.^51,52^ The prevalence of trauma and combat exposure among women who routinely use VA primary care reinforces the need to implement trauma-informed care across multiple clinical settings and optimize feasible options, including trauma-informed telehealth.^53–55^ VA has announced its broad support for trauma-informed care; implementation will require additional consultation time, provider training, effective mental health referral mechanisms, and attention to trust building within provider-patient relationships.^56,57^

Medical complexity among women who routinely use VA primary care is underscored by the prevalence of co-occurring chronic disease and mental health issues. Prior research has established links between mental health issues and chronic disease. For example, PTSD is a risk factor for developing heart disease and other chronic illnesses, and patients with anxiety or depression concurrent with chronic physical illness report more chronic disease symptoms than those with chronic disease alone.^58–61^ This suggests that accurate mental health diagnosis and care among women Veterans with chronic illness is key to understanding and managing chronic disease symptom burden.

Routine users of VA primary care report satisfaction with VA women’s health services have confidence in their providers and would recommend VA to other women Veterans. However, perception of the quality of women’s care provided in VA varies by age; a substantial minority of women in the youngest and middle age groups perceived women’s care provided outside VA as better than care provided inside. This perception warrants future study and continued investment in quality improvement. Women’s preference for receiving care in women-only settings and from female providers is evident. This aligns with previous research indicating that, within VA, quality of women’s care and satisfaction is higher among patients seen by designated women’s health providers and in women’s clinics.^62,63^ Women’s preferences should continue to influence facility planning.

Examining our results by age, we note distinct and varying health needs across the lifespan of women Veterans who routinely use VA primary care. For example, among women aged 18–44, the prevalence of elevated cholesterol (15%) is substantially higher than among the same age group of women in the general population (7.2%) and indicates a need for increased early prevention efforts. Additionally, women aged 18–44 had more combat exposure than women aged 45–65+. This high level of exposure is likely to continue now that all military positions, including active combat, are open to women. This may result in a need for increased deployment-related support, such as PTSD treatment, tailored to women. In addition, access to comprehensive, high-quality, and well-coordinated reproductive healthcare, including maternity care, is critical to women aged 18–44.

When looking at women aged 45–64, several important findings emerge. Concurrent physical and mental health issues peak in this age group. Women in this group most commonly report that pain interferes with their work “a lot/all” of the time, highlighting need for effective pain management across a large volume of women Veteran VA users. Also, sexual trauma is most prevalent among women aged 45–64, reinforcing the need for convenient access to mental health services and trauma-informed specialty care as women increase engagement in chronic disease (e.g., cardiovascular) care. Lastly, access to menopause-related care is critical in this age group.

Among women aged 65+, the prevalence of chronic illness mounts, requiring intensified management. The median medical comorbidity score (SIC) for women aged 65+ (7) is nearly twice that identified by DeSalvo (2005) among men VA users of the same age (4).^64^ Although prevalence of positive mental health screens is lowest among women aged 65+, almost one-third do screen positive, highlighting the need for elder care that focuses on mental healthcare in addition to chronic disease management.

Our study represents the first population-based survey of women Veterans who are VA routine primary care users. However, it does have limitations. To inform care delivery, provider training, and care coordination for women who rely on VA primary care, our sample includes only women with 3+ prior year primary care visits. While this visit frequency is higher than among insured women in the general public, prior research has demonstrated that women Veterans using VA services have greater health needs compared to non-Veteran women. ^1,31,65^ Our findings therefore do not represent women who do not routinely use VA primary care and may not inform service delivery in other settings. We used self-report measures, which are subject to recall and social desirability bias. However, self-report may also illuminate previously unreported symptoms, conditions, or experiences. Additionally, while we have data regarding model(s) of care available at each facility, we do not know the specific care model within which each participant received care. This limitation may undermine our ability to interpret some aspects of satisfaction. These limitations are balanced by strengths. The sample included diverse women from twelve VA sites representing different geographical regions, resource levels, complexity, and sizes. Our weighting procedures also improve sample generalizability and utility of our findings for informing practice and policy within VA.

This is the first paper to characterize healthcare needs, utilization patterns, and preferences among women Veterans who routinely use VA primary care services. Our findings highlight the importance of continued investment in women’s health clinical expertise and infrastructure within VA. They should inform ongoing improvements in women Veterans’ access to comprehensive services tailored to their needs at different stages of their lives. Continuing improvement will require a primary care delivery system capable of responding to the needs, utilization patterns, and preferences of women who rely on VA care, including sensitivity to their gender-specific and trauma-related needs.

## Data Availability

The datasets generated during and/or analyzed during the current study are not publicly available because patient consent was not obtained to do so. Additionally, women Veterans are a small enough subset of all Veterans that the survey data could be used to re-identify participants, which would run counter to VA human subjects protections requirements.
